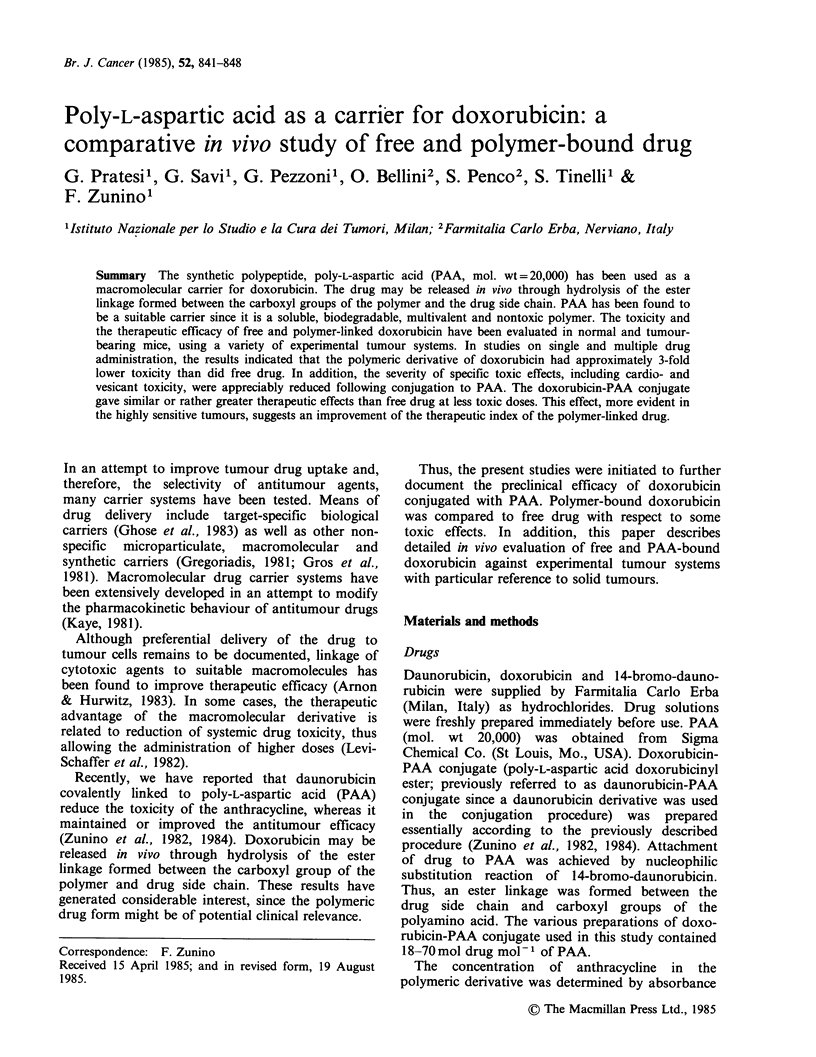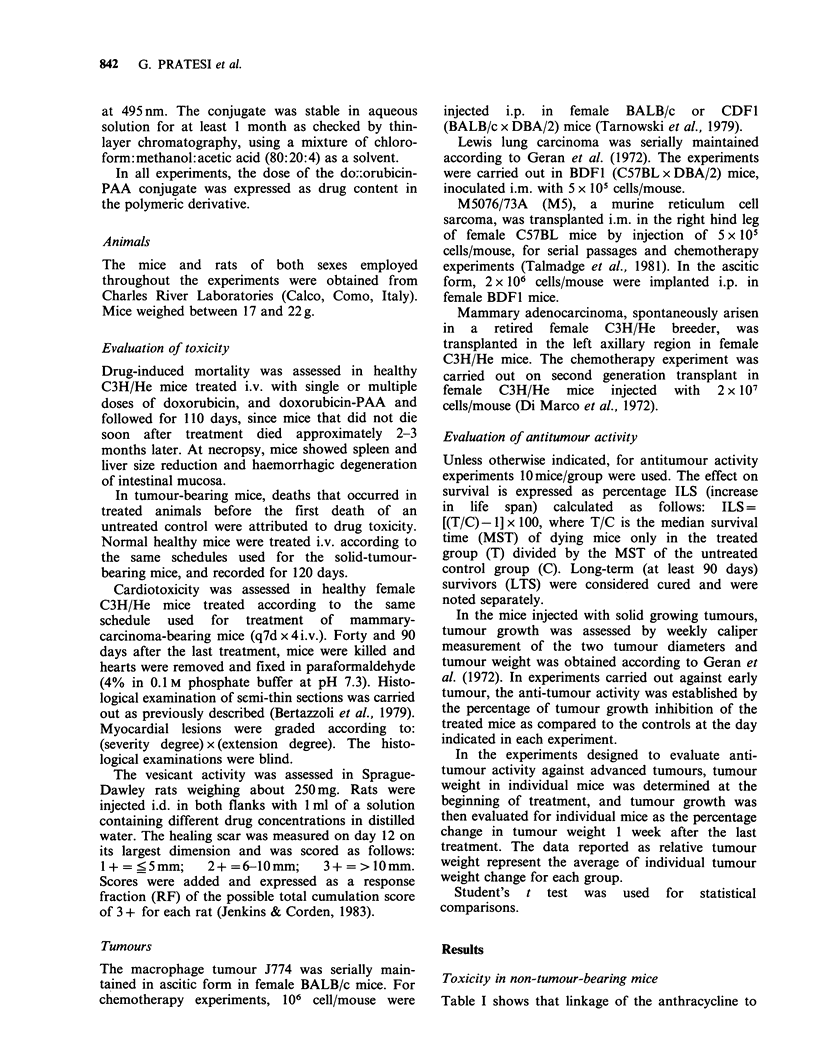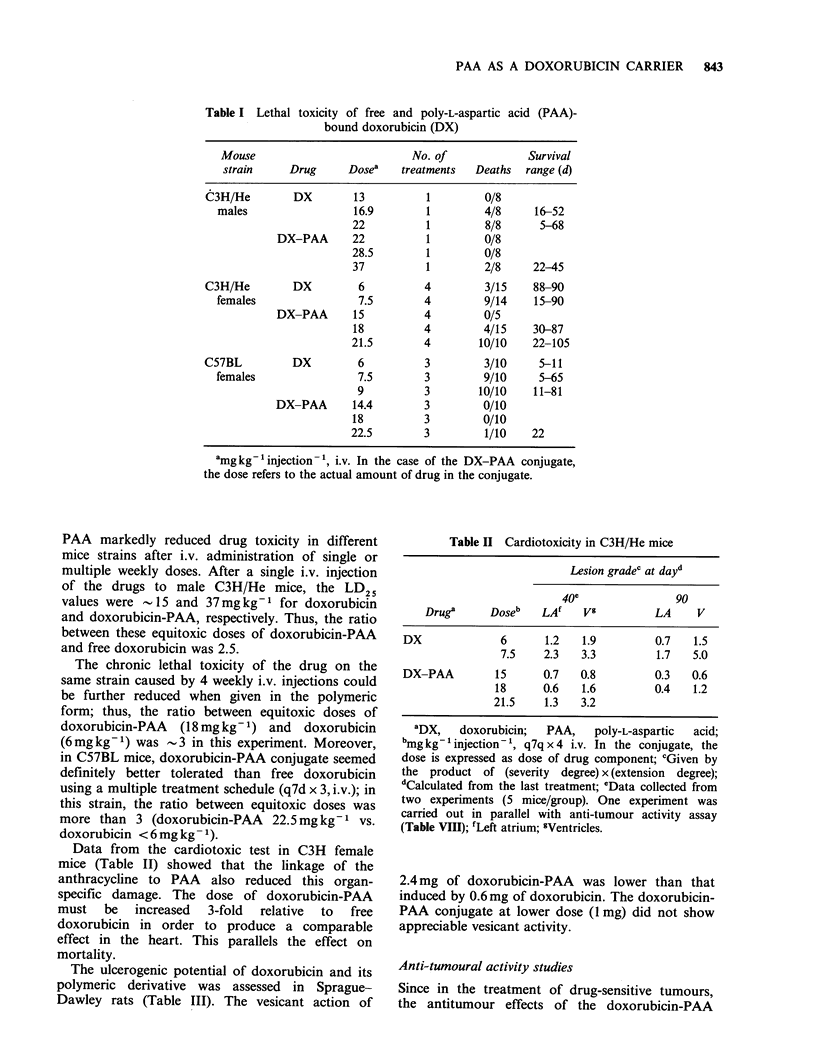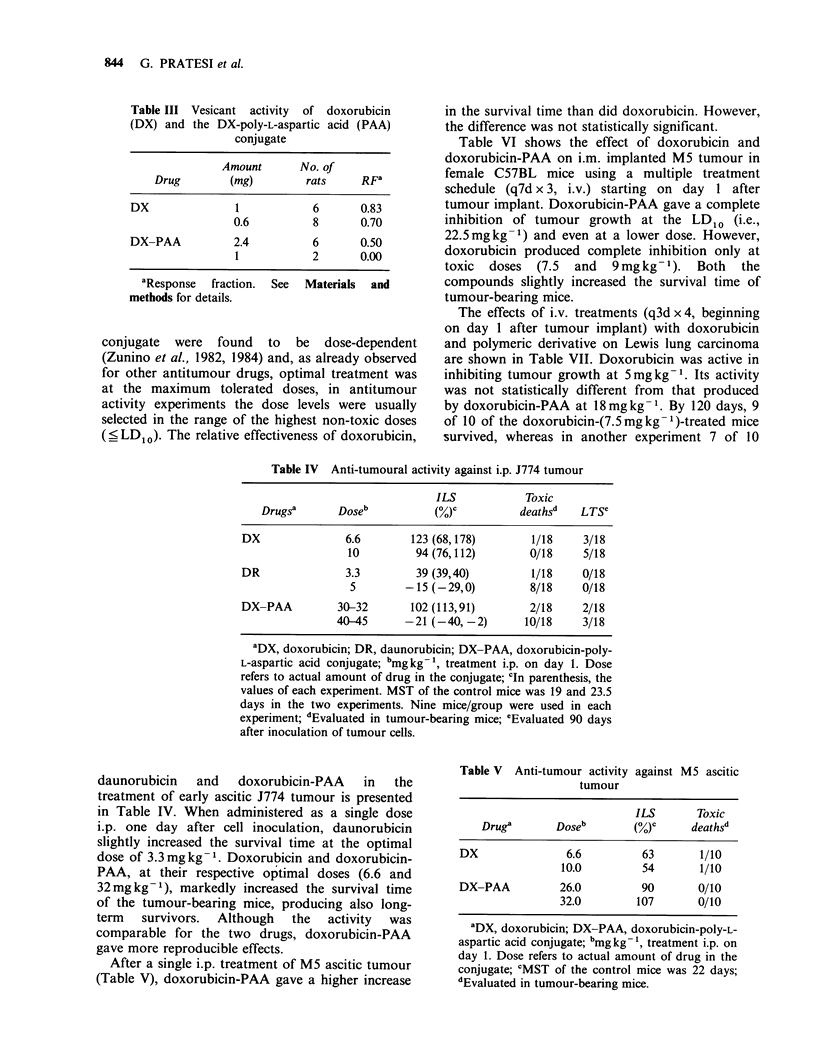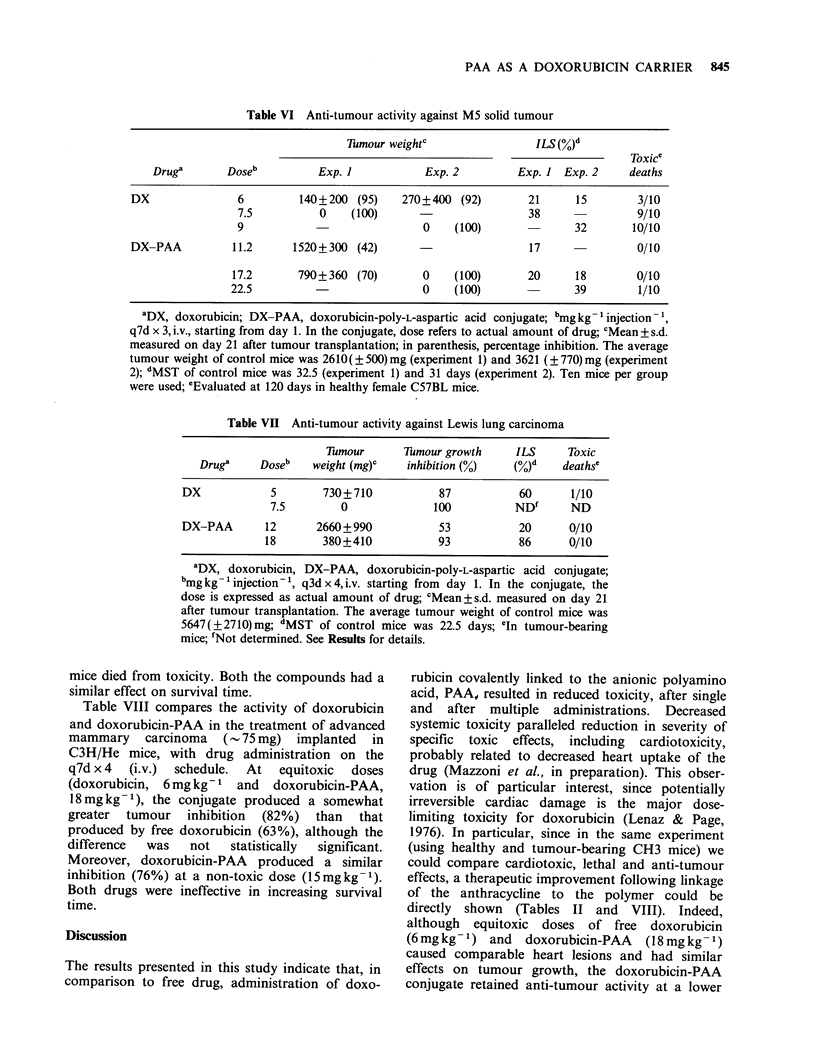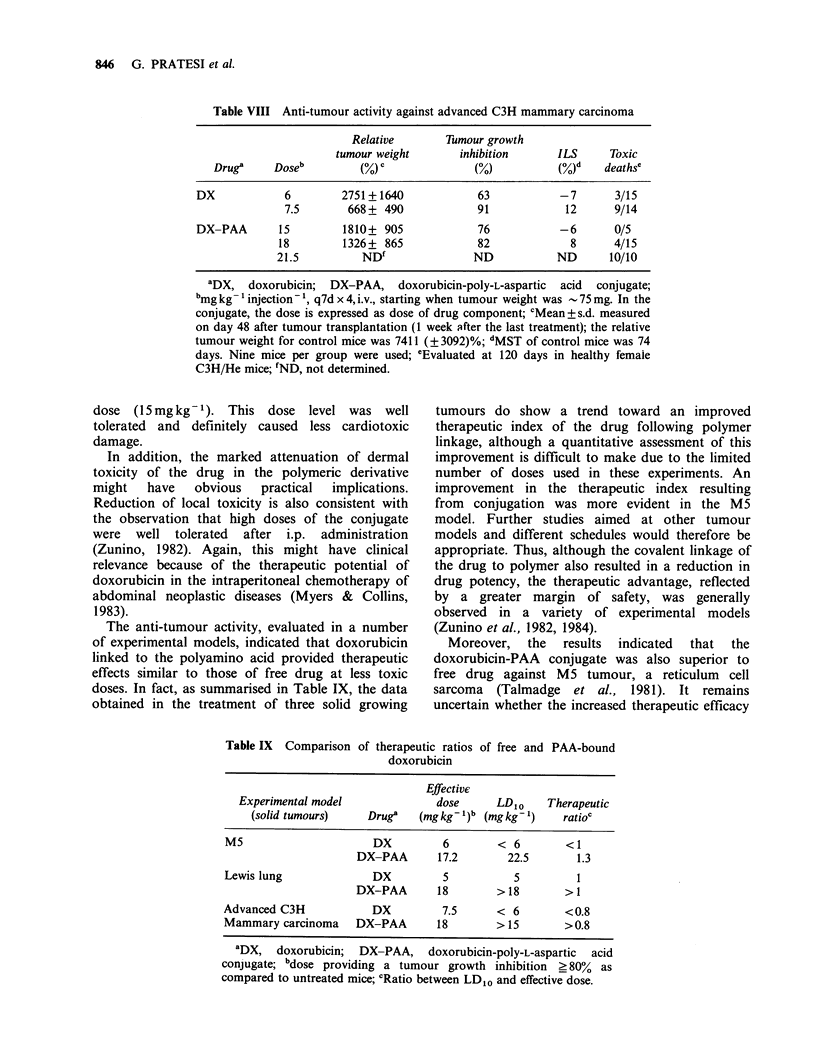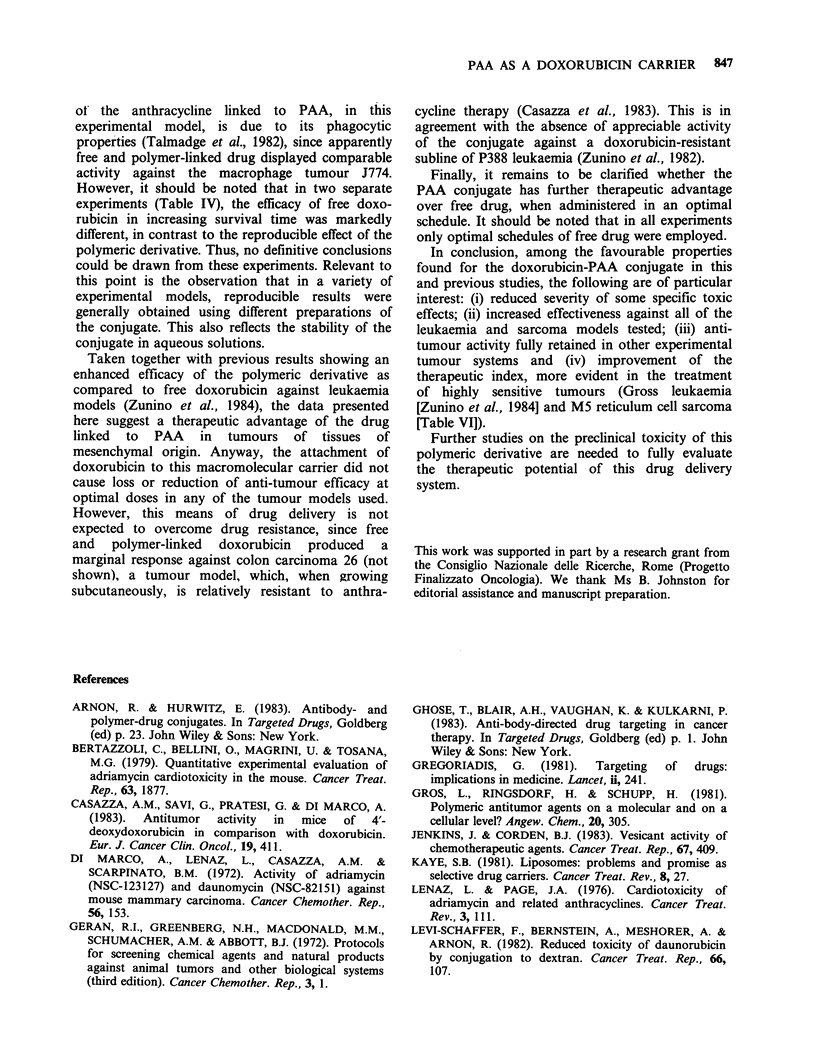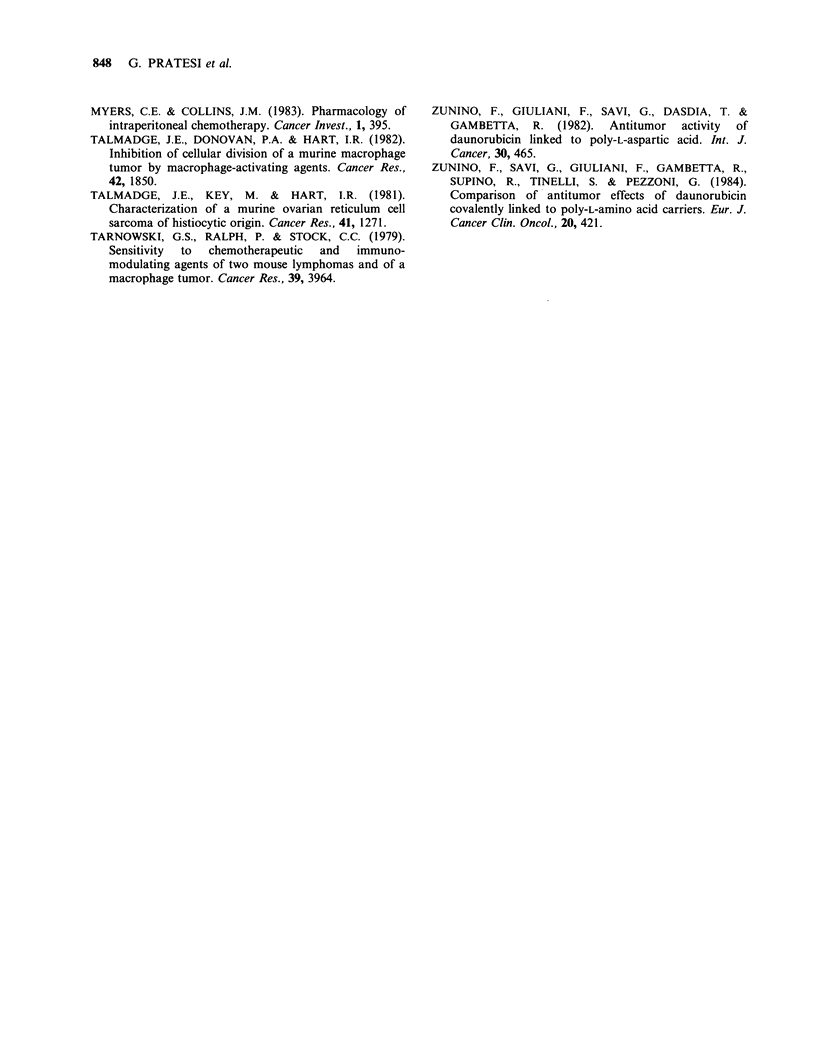# Poly-L-aspartic acid as a carrier for doxorubicin: a comparative in vivo study of free and polymer-bound drug.

**DOI:** 10.1038/bjc.1985.267

**Published:** 1985-12

**Authors:** G. Pratesi, G. Savi, G. Pezzoni, O. Bellini, S. Penco, S. Tinelli, F. Zunino

## Abstract

The synthetic polypeptide, poly-L-aspartic acid (PAA, mol. wt = 20,000) has been used as a macromolecular carrier for doxorubicin. The drug may be released in vivo through hydrolysis of the ester linkage formed between the carboxyl groups of the polymer and the drug side chain. PAA has been found to be a suitable carrier since it is a soluble, biodegradable, multivalent and nontoxic polymer. The toxicity and the therapeutic efficacy of free and polymer-linked doxorubicin have been evaluated in normal and tumour-bearing mice, using a variety of experimental tumour systems. In studies on single and multiple drug administration, the results indicated that the polymeric derivative of doxorubicin had approximately 3-fold lower toxicity than did free drug. In addition, the severity of specific toxic effects, including cardio- and vesicant toxicity, were appreciably reduced following conjugation to PAA. The doxorubicin-PAA conjugate gave similar or rather greater therapeutic effects than free drug at less toxic doses. This effect, more evident in the highly sensitive tumours, suggests an improvement of the therapeutic index of the polymer-linked drug.


*Br. J. Cancer* (1985), **52**, 841–848

### Poly-L-aspartic acid as a carrier for doxorubicin: a comparative *in vivo* study of free and polymer-bound drug

G. Pratesi[1], G. Savi[1], G. Pezzoni[1], O. Bellini[2], S. Penco[2], S. Tinelli[1] & F. Zunino[1]

[1]*Istituto Nazionale per lo Studio e la Cura dei Tumori, Milan;* [2]*Farmitalia Carlo Erba, Nerviano, Italy*

**Summary** The synthetic polypeptide, poly-L-aspartic acid (PAA, mol. wt = 20,000) has been used as a macromolecular carrier for doxorubicin. The drug may be released *in vivo* through hydrolysis of the ester linkage formed between the carboxyl groups of the polymer and the drug side chain. PAA has been found to be a suitable carrier since it is a soluble, biodegradable, multivalent and nontoxic polymer. The toxicity and the therapeutic efficacy of free and polymer-linked doxorubicin have been evaluated in normal and tumour-bearing mice, using a variety of experimental tumour systems. In studies on single and multiple drug administration, the results indicated that the polymeric derivative of doxorubicin had approximately 3-fold lower toxicity than did free drug. In addition, the severity of specific toxic effects, including cardio- and vesicant toxicity, were appreciably reduced following conjugation to PAA. The doxorubicin-PAA conjugate gave similar or rather greater therapeutic effects than free drug at less toxic doses. This effect, more evident in the highly sensitive tumours, suggests an improvement of the therapeutic index of the polymer-linked drug.

In an attempt to improve tumour drug uptake and, therefore, the selectivity of antitumour agents, many carrier systems have been tested. Means of drug delivery include target-specific biological carriers (Ghose *et al.*, 1983) as well as other non-specific microparticulate, macromolecular and synthetic carriers (Gregoriadis, 1981; Gros *et al.*, 1981). Macromolecular drug carrier systems have been extensively developed in an attempt to modify the pharmacokinetic behaviour of antitumour drugs (Kaye, 1981).

Although preferential delivery of the drug to tumour cells remains to be documented, linkage of cytotoxic agents to suitable macromolecules has been found to improve therapeutic efficacy (Arnon & Hurwitz, 1983). In some cases, the therapeutic advantage of the macromolecular derivative is related to reduction of systemic drug toxicity, thus allowing the administration of higher doses (Levi-Schaffer *et al.*, 1982).

Recently, we have reported that daunorubicin covalently linked to poly-L-aspartic acid (PAA) reduce the toxicity of the anthracycline, whereas it maintained or improved the antitumour efficacy (Zunino *et al.*, 1982, 1984). Doxorubicin may be released *in vivo* through hydrolysis of the ester linkage formed between the carboxyl group of the polymer and drug side chain. These results have generated considerable interest, since the polymeric drug form might be of potential clinical relevance.

Correspondence: F. Zunino
Received 15 April 1985; and in revised form, 19 August 1985.

Thus, the present studies were initiated to further document the preclinical efficacy of doxorubicin conjugated with PAA. Polymer-bound doxorubicin was compared to free drug with respect to some toxic effects. In addition, this paper describes detailed *in vivo* evaluation of free and PAA-bound doxorubicin against experimental tumour systems with particular reference to solid tumours.

### Materials and methods

### *Drugs*

Daunorubicin, doxorubicin and 14-bromo-daunorubicin were supplied by Farmitalia Carlo Erba (Milan, Italy) as hydrochlorides. Drug solutions were freshly prepared immediately before use. PAA (mol. wt 20,000) was obtained from Sigma Chemical Co. (St Louis, Mo., USA). Doxorubicin-PAA conjugate (poly-L-aspartic acid doxorubicinyl ester; previously referred to as daunorubicin-PAA conjugate since a daunorubicin derivative was used in the conjugation procedure) was prepared essentially according to the previously described procedure (Zunino *et al.*, 1982, 1984). Attachment of drug to PAA was achieved by nucleophilic substitution reaction of 14-bromo-daunorubicin. Thus, an ester linkage was formed between the drug side chain and carboxyl groups of the polyamino acid. The various preparations of doxorubicin-PAA conjugate used in this study contained 18–70 mol drug $mol^{-1}$ of PAA.

The concentration of anthracycline in the polymeric derivative was determined by absorbance

© The Macmillan Press Ltd., 1985

at 495 nm. The conjugate was stable in aqueous solution for at least 1 month as checked by thin-layer chromatography, using a mixture of chloroform:methanol:acetic acid (80:20:4) as a solvent.

In all experiments, the dose of the doxorubicin-PAA conjugate was expressed as drug content in the polymeric derivative.

*Animals*

The mice and rats of both sexes employed throughout the experiments were obtained from Charles River Laboratories (Calco, Como, Italy). Mice weighed between 17 and 22 g.

*Evaluation of toxicity*

Drug-induced mortality was assessed in healthy C3H/He mice treated i.v. with single or multiple doses of doxorubicin, and doxorubicin-PAA and followed for 110 days, since mice that did not die soon after treatment died approximately 2–3 months later. At necropsy, mice showed spleen and liver size reduction and haemorrhagic degeneration of intestinal mucosa.

In tumour-bearing mice, deaths that occurred in treated animals before the first death of an untreated control were attributed to drug toxicity. Normal healthy mice were treated i.v. according to the same schedules used for the solid-tumour-bearing mice, and recorded for 120 days.

Cardiotoxicity was assessed in healthy female C3H/He mice treated according to the same schedule used for treatment of mammary-carcinoma-bearing mice (q7d × 4 i.v.). Forty and 90 days after the last treatment, mice were killed and hearts were removed and fixed in paraformaldehyde (4% in 0.1 M phosphate buffer at pH 7.3). Histological examination of semi-thin sections was carried out as previously described (Bertazzoli et al., 1979). Myocardial lesions were graded according to: (severity degree) × (extension degree). The histological examinations were blind.

The vesicant activity was assessed in Sprague-Dawley rats weighing about 250 mg. Rats were injected i.d. in both flanks with 1 ml of a solution containing different drug concentrations in distilled water. The healing scar was measured on day 12 on its largest dimension and was scored as follows: 1+ = ≦5 mm; 2+ = 6–10 mm; 3+ = > 10 mm. Scores were added and expressed as a response fraction (RF) of the possible total cumulation score of 3+ for each rat (Jenkins & Corden, 1983).

*Tumours*

The macrophage tumour J774 was serially maintained in ascitic form in female BALB/c mice. For chemotherapy experiments, $10^6$ cell/mouse were injected i.p. in female BALB/c or CDF1 (BALB/c × DBA/2) mice (Tarnowski et al., 1979).

Lewis lung carcinoma was serially maintained according to Geran et al. (1972). The experiments were carried out in BDF1 (C57BL × DBA/2) mice, inoculated i.m. with $5 \times 10^5$ cells/mouse.

M5076/73A (M5), a murine reticulum cell sarcoma, was transplanted i.m. in the right hind leg of female C57BL mice by injection of $5 \times 10^5$ cells/mouse, for serial passages and chemotherapy experiments (Talmadge et al., 1981). In the ascitic form, $2 \times 10^6$ cells/mouse were implanted i.p. in female BDF1 mice.

Mammary adenocarcinoma, spontaneously arisen in a retired female C3H/He breeder, was transplanted in the left axillary region in female C3H/He mice. The chemotherapy experiment was carried out on second generation transplant in female C3H/He mice injected with $2 \times 10^7$ cells/mouse (Di Marco et al., 1972).

*Evaluation of antitumour activity*

Unless otherwise indicated, for antitumour activity experiments 10 mice/group were used. The effect on survival is expressed as percentage ILS (increase in life span) calculated as follows: ILS = [(T/C) − 1] × 100, where T/C is the median survival time (MST) of dying mice only in the treated group (T) divided by the MST of the untreated control group (C). Long-term (at least 90 days) survivors (LTS) were considered cured and were noted separately.

In the mice injected with solid growing tumours, tumour growth was assessed by weekly caliper measurement of the two tumour diameters and tumour weight was obtained according to Geran et al. (1972). In experiments carried out against early tumour, the anti-tumour activity was established by the percentage of tumour growth inhibition of the treated mice as compared to the controls at the day indicated in each experiment.

In the experiments designed to evaluate anti-tumour activity against advanced tumours, tumour weight in individual mice was determined at the beginning of treatment, and tumour growth was then evaluated for individual mice as the percentage change in tumour weight 1 week after the last treatment. The data reported as relative tumour weight represent the average of individual tumour weight change for each group.

Student's *t* test was used for statistical comparisons.

### Results

*Toxicity in non-tumour-bearing mice*

Table I shows that linkage of the anthracycline to

**Table I** Lethal toxicity of free and poly-L-aspartic acid (PAA)-bound doxorubicin (DX)

| *Mouse strain* | *Drug* | *Dose*[a] | *No. of treatments* | *Deaths* | *Survival range (d)* |
|---|---|---|---|---|---|
| C3H/He males | DX | 13 | 1 | 0/8 | |
| | | 16.9 | 1 | 4/8 | 16–52 |
| | | 22 | 1 | 8/8 | 5–68 |
| | DX–PAA | 22 | 1 | 0/8 | |
| | | 28.5 | 1 | 0/8 | |
| | | 37 | 1 | 2/8 | 22–45 |
| C3H/He females | DX | 6 | 4 | 3/15 | 88–90 |
| | | 7.5 | 4 | 9/14 | 15–90 |
| | DX–PAA | 15 | 4 | 0/5 | |
| | | 18 | 4 | 4/15 | 30–87 |
| | | 21.5 | 4 | 10/10 | 22–105 |
| C57BL females | DX | 6 | 3 | 3/10 | 5–11 |
| | | 7.5 | 3 | 9/10 | 5–65 |
| | | 9 | 3 | 10/10 | 11–81 |
| | DX–PAA | 14.4 | 3 | 0/10 | |
| | | 18 | 3 | 0/10 | |
| | | 22.5 | 3 | 1/10 | 22 |

[a]$mg\,kg^{-1}\,injection^{-1}$, i.v. In the case of the DX–PAA conjugate, the dose refers to the actual amount of drug in the conjugate.

PAA markedly reduced drug toxicity in different mice strains after i.v. administration of single or multiple weekly doses. After a single i.v. injection of the drugs to male C3H/He mice, the $LD_{25}$ values were ~15 and 37 $mg\,kg^{-1}$ for doxorubicin and doxorubicin-PAA, respectively. Thus, the ratio between these equitoxic doses of doxorubicin-PAA and free doxorubicin was 2.5.

The chronic lethal toxicity of the drug on the same strain caused by 4 weekly i.v. injections could be further reduced when given in the polymeric form; thus, the ratio between equitoxic doses of doxorubicin-PAA (18 $mg\,kg^{-1}$) and doxorubicin (6 $mg\,kg^{-1}$) was ~3 in this experiment. Moreover, in C57BL mice, doxorubicin-PAA conjugate seemed definitely better tolerated than free doxorubicin using a multiple treatment schedule (q7d × 3, i.v.); in this strain, the ratio between equitoxic doses was more than 3 (doxorubicin-PAA 22.5 $mg\,kg^{-1}$ vs. doxorubicin < 6 $mg\,kg^{-1}$).

Data from the cardiotoxic test in C3H female mice (Table II) showed that the linkage of the anthracycline to PAA also reduced this organ-specific damage. The dose of doxorubicin-PAA must be increased 3-fold relative to free doxorubicin in order to produce a comparable effect in the heart. This parallels the effect on mortality.

The ulcerogenic potential of doxorubicin and its polymeric derivative was assessed in Sprague–Dawley rats (Table III). The vesicant action of 2.4 mg of doxorubicin-PAA was lower than that induced by 0.6 mg of doxorubicin. The doxorubicin-PAA conjugate at lower dose (1 mg) did not show appreciable vesicant activity.

**Table II** Cardiotoxicity in C3H/He mice

| | | *Lesion grade*[c] *at day*[d] | | | |
|---|---|---|---|---|---|
| | | *40*[e] | | *90* | |
| *Drug*[a] | *Dose*[b] | *LA*[f] | *V*[g] | *LA* | *V* |
| DX | 6 | 1.2 | 1.9 | 0.7 | 1.5 |
| | 7.5 | 2.3 | 3.3 | 1.7 | 5.0 |
| DX–PAA | 15 | 0.7 | 0.8 | 0.3 | 0.6 |
| | 18 | 0.6 | 1.6 | 0.4 | 1.2 |
| | 21.5 | 1.3 | 3.2 | | |

[a]DX, doxorubicin; PAA, poly-L-aspartic acid; [b]$mg\,kg^{-1}\,injection^{-1}$, q7q × 4 i.v. In the conjugate, the dose is expressed as dose of drug component; [c]Given by the product of (severity degree) × (extension degree); [d]Calculated from the last treatment; [e]Data collected from two experiments (5 mice/group). One experiment was carried out in parallel with anti-tumour activity assay (**Table VIII**); [f]Left atrium; [g]Ventricles.

### *Anti-tumoural activity studies*

Since in the treatment of drug-sensitive tumours, the antitumour effects of the doxorubicin-PAA

Table III Vesicant activity of doxorubicin (DX) and the DX-poly-L-aspartic acid (PAA) conjugate

| Drug | Amount (mg) | No. of rats | RF[a] |
|---|---|---|---|
| DX | 1 | 6 | 0.83 |
| | 0.6 | 8 | 0.70 |
| DX–PAA | 2.4 | 6 | 0.50 |
| | 1 | 2 | 0.00 |

[a]Response fraction. See **Materials and methods** for details.

conjugate were found to be dose-dependent (Zunino *et al.*, 1982, 1984) and, as already observed for other antitumour drugs, optimal treatment was at the maximum tolerated doses, in antitumour activity experiments the dose levels were usually selected in the range of the highest non-toxic doses ($\leqq LD_{10}$). The relative effectiveness of doxorubicin, daunorubicin and doxorubicin-PAA in the treatment of early ascitic J774 tumour is presented in Table IV. When administered as a single dose i.p. one day after cell inoculation, daunorubicin slightly increased the survival time at the optimal dose of $3.3\,mg\,kg^{-1}$. Doxorubicin and doxorubicin-PAA, at their respective optimal doses (6.6 and $32\,mg\,kg^{-1}$), markedly increased the survival time of the tumour-bearing mice, producing also long-term survivors. Although the activity was comparable for the two drugs, doxorubicin-PAA gave more reproducible effects.

After a single i.p. treatment of M5 ascitic tumour (Table V), doxorubicin-PAA gave a higher increase in the survival time than did doxorubicin. However, the difference was not statistically significant.

Table VI shows the effect of doxorubicin and doxorubicin-PAA on i.m. implanted M5 tumour in female C57BL mice using a multiple treatment schedule (q7d × 3, i.v.) starting on day 1 after tumour implant. Doxorubicin-PAA gave a complete inhibition of tumour growth at the $LD_{10}$ (i.e., $22.5\,mg\,kg^{-1}$) and even at a lower dose. However, doxorubicin produced complete inhibition only at toxic doses (7.5 and $9\,mg\,kg^{-1}$). Both the compounds slightly increased the survival time of tumour-bearing mice.

The effects of i.v. treatments (q3d × 4, beginning on day 1 after tumour implant) with doxorubicin and polymeric derivative on Lewis lung carcinoma are shown in Table VII. Doxorubicin was active in inhibiting tumour growth at $5\,mg\,kg^{-1}$. Its activity was not statistically different from that produced by doxorubicin-PAA at $18\,mg\,kg^{-1}$. By 120 days, 9 of 10 of the doxorubicin-($7.5\,mg\,kg^{-1}$)-treated mice survived, whereas in another experiment 7 of 10

Table IV Anti-tumoural activity against i.p. J774 tumour

| Drugs[a] | Dose[b] | ILS (%)[c] | Toxic deaths[d] | LTS[e] |
|---|---|---|---|---|
| DX | 6.6 | 123 (68, 178) | 1/18 | 3/18 |
| | 10 | 94 (76, 112) | 0/18 | 5/18 |
| DR | 3.3 | 39 (39, 40) | 1/18 | 0/18 |
| | 5 | −15 (−29, 0) | 8/18 | 0/18 |
| DX–PAA | 30–32 | 102 (113, 91) | 2/18 | 2/18 |
| | 40–45 | −21 (−40, −2) | 10/18 | 3/18 |

[a]DX, doxorubicin; DR, daunorubicin; DX–PAA, doxorubicin-poly-L-aspartic acid conjugate; [b]$mg\,kg^{-1}$, treatment i.p. on day 1. Dose refers to actual amount of drug in the conjugate; [c]In parenthesis, the values of each experiment. MST of the control mice was 19 and 23.5 days in the two experiments. Nine mice/group were used in each experiment; [d]Evaluated in tumour-bearing mice; [e]Evaluated 90 days after inoculation of tumour cells.

Table V Anti-tumour activity against M5 ascitic tumour

| Drug[a] | Dose[b] | ILS (%)[c] | Toxic deaths[d] |
|---|---|---|---|
| DX | 6.6 | 63 | 1/10 |
| | 10.0 | 54 | 1/10 |
| DX–PAA | 26.0 | 90 | 0/10 |
| | 32.0 | 107 | 0/10 |

[a]DX, doxorubicin; DX–PAA, doxorubicin-poly-L-aspartic acid conjugate; [b]$mg\,kg^{-1}$, treatment i.p. on day 1. Dose refers to actual amount of drug in the conjugate; [c]MST of the control mice was 22 days; [d]Evaluated in tumour-bearing mice.

**Table VI** Anti-tumour activity against M5 solid tumour

| *Drug*[a] | *Dose*[b] | *Tumour weight*[c] *Exp. 1* | *Tumour weight*[c] *Exp. 2* | *ILS (%)*[d] *Exp. 1* | *ILS (%)*[d] *Exp. 2* | *Toxic*[e] *deaths* |
|---|---|---|---|---|---|---|
| DX | 6 | 140±200 (95) | 270±400 (92) | 21 | 15 | 3/10 |
| | 7.5 | 0 (100) | — | 38 | — | 9/10 |
| | 9 | — | 0 (100) | — | 32 | 10/10 |
| DX–PAA | 11.2 | 1520±300 (42) | — | 17 | — | 0/10 |
| | 17.2 | 790±360 (70) | 0 (100) | 20 | 18 | 0/10 |
| | 22.5 | — | 0 (100) | — | 39 | 1/10 |

[a]DX, doxorubicin; DX–PAA, doxorubicin-poly-L-aspartic acid conjugate; [b]$mg\,kg^{-1}$ $injection^{-1}$, q7d × 3, i.v., starting from day 1. In the conjugate, dose refers to actual amount of drug; [c]Mean ± s.d. measured on day 21 after tumour transplantation; in parenthesis, percentage inhibition. The average tumour weight of control mice was 2610 (±500) mg (experiment 1) and 3621 (±770) mg (experiment 2); [d]MST of control mice was 32.5 (experiment 1) and 31 days (experiment 2). Ten mice per group were used; [e]Evaluated at 120 days in healthy female C57BL mice.

**Table VII** Anti-tumour activity against Lewis lung carcinoma

| *Drug*[a] | *Dose*[b] | *Tumour weight (mg)*[c] | *Tumour growth inhibition (%)* | *ILS (%)*[d] | *Toxic deaths*[e] |
|---|---|---|---|---|---|
| DX | 5 | 730±710 | 87 | 60 | 1/10 |
| | 7.5 | 0 | 100 | ND[f] | ND |
| DX–PAA | 12 | 2660±990 | 53 | 20 | 0/10 |
| | 18 | 380±410 | 93 | 86 | 0/10 |

[a]DX, doxorubicin, DX–PAA, doxorubicin-poly-L-aspartic acid conjugate; [b]$mg\,kg^{-1}$ $injection^{-1}$, q3d × 4, i.v. starting from day 1. In the conjugate, the dose is expressed as actual amount of drug; [c]Mean ± s.d. measured on day 21 after tumour transplantation. The average tumour weight of control mice was 5647 (±2710) mg; [d]MST of control mice was 22.5 days; [e]In tumour-bearing mice; [f]Not determined. See **Results** for details.

mice died from toxicity. Both the compounds had a similar effect on survival time.

Table VIII compares the activity of doxorubicin and doxorubicin-PAA in the treatment of advanced mammary carcinoma (~75 mg) implanted in C3H/He mice, with drug administration on the q7d × 4 (i.v.) schedule. At equitoxic doses (doxorubicin, $6\,mg\,kg^{-1}$ and doxorubicin-PAA, $18\,mg\,kg^{-1}$), the conjugate produced a somewhat greater tumour inhibition (82%) than that produced by free doxorubicin (63%), although the difference was not statistically significant. Moreover, doxorubicin-PAA produced a similar inhibition (76%) at a non-toxic dose ($15\,mg\,kg^{-1}$). Both drugs were ineffective in increasing survival time.

### Discussion

The results presented in this study indicate that, in comparison to free drug, administration of doxorubicin covalently linked to the anionic polyamino acid, PAA, resulted in reduced toxicity, after single and after multiple administrations. Decreased systemic toxicity paralleled reduction in severity of specific toxic effects, including cardiotoxicity, probably related to decreased heart uptake of the drug (Mazzoni *et al.*, in preparation). This observation is of particular interest, since potentially irreversible cardiac damage is the major dose-limiting toxicity for doxorubicin (Lenaz & Page, 1976). In particular, since in the same experiment (using healthy and tumour-bearing CH3 mice) we could compare cardiotoxic, lethal and anti-tumour effects, a therapeutic improvement following linkage of the anthracycline to the polymer could be directly shown (Tables II and VIII). Indeed, although equitoxic doses of free doxorubicin ($6\,mg\,kg^{-1}$) and doxorubicin-PAA ($18\,mg\,kg^{-1}$) caused comparable heart lesions and had similar effects on tumour growth, the doxorubicin-PAA conjugate retained anti-tumour activity at a lower

**Table VIII** Anti-tumour activity against advanced C3H mammary carcinoma

| *Drug*[a] | *Dose*[b] | *Relative tumour weight* (%)[c] | *Tumour growth inhibition* (%) | *ILS* (%)[d] | *Toxic deaths*[e] |
|---|---|---|---|---|---|
| DX | 6 | 2751 ± 1640 | 63 | −7 | 3/15 |
| | 7.5 | 668 ± 490 | 91 | 12 | 9/14 |
| DX–PAA | 15 | 1810 ± 905 | 76 | −6 | 0/5 |
| | 18 | 1326 ± 865 | 82 | 8 | 4/15 |
| | 21.5 | ND[f] | ND | ND | 10/10 |

[a]DX, doxorubicin; DX–PAA, doxorubicin-poly-L-aspartic acid conjugate; [b]$mg\,kg^{-1}$ $injection^{-1}$, q7d × 4, i.v., starting when tumour weight was ~75 mg. In the conjugate, the dose is expressed as dose of drug component; [c]Mean ± s.d. measured on day 48 after tumour transplantation (1 week after the last treatment); the relative tumour weight for control mice was 7411 (±3092)%; [d]MST of control mice was 74 days. Nine mice per group were used; [e]Evaluated at 120 days in healthy female C3H/He mice; [f]ND, not determined.

dose ($15\,mg\,kg^{-1}$). This dose level was well tolerated and definitely caused less cardiotoxic damage.

In addition, the marked attenuation of dermal toxicity of the drug in the polymeric derivative might have obvious practical implications. Reduction of local toxicity is also consistent with the observation that high doses of the conjugate were well tolerated after i.p. administration (Zunino, 1982). Again, this might have clinical relevance because of the therapeutic potential of doxorubicin in the intraperitoneal chemotherapy of abdominal neoplastic diseases (Myers & Collins, 1983).

The anti-tumour activity, evaluated in a number of experimental models, indicated that doxorubicin linked to the polyamino acid provided therapeutic effects similar to those of free drug at less toxic doses. In fact, as summarised in Table IX, the data obtained in the treatment of three solid growing tumours do show a trend toward an improved therapeutic index of the drug following polymer linkage, although a quantitative assessment of this improvement is difficult to make due to the limited number of doses used in these experiments. An improvement in the therapeutic index resulting from conjugation was more evident in the M5 model. Further studies aimed at other tumour models and different schedules would therefore be appropriate. Thus, although the covalent linkage of the drug to polymer also resulted in a reduction in drug potency, the therapeutic advantage, reflected by a greater margin of safety, was generally observed in a variety of experimental models (Zunino *et al.*, 1982, 1984).

Moreover, the results indicated that the doxorubicin-PAA conjugate was also superior to free drug against M5 tumour, a reticulum cell sarcoma (Talmadge *et al.*, 1981). It remains uncertain whether the increased therapeutic efficacy

**Table IX** Comparison of therapeutic ratios of free and PAA-bound doxorubicin

| *Experimental model (solid tumours)* | *Drug*[a] | *Effective dose* ($mg\,kg^{-1}$)[b] | $LD_{10}$ ($mg\,kg^{-1}$) | *Therapeutic ratio*[c] |
|---|---|---|---|---|
| M5 | DX | 6 | < 6 | < 1 |
| | DX–PAA | 17.2 | 22.5 | 1.3 |
| Lewis lung | DX | 5 | 5 | 1 |
| | DX–PAA | 18 | > 18 | > 1 |
| Advanced C3H | DX | 7.5 | < 6 | < 0.8 |
| Mammary carcinoma | DX–PAA | 18 | > 15 | > 0.8 |

[a]DX, doxorubicin; DX–PAA, doxorubicin-poly-L-aspartic acid conjugate; [b]dose providing a tumour growth inhibition ≥80% as compared to untreated mice; [c]Ratio between $LD_{10}$ and effective dose.

of the anthracycline linked to PAA, in this experimental model, is due to its phagocytic properties (Talmadge *et al.*, 1982), since apparently free and polymer-linked drug displayed comparable activity against the macrophage tumour J774. However, it should be noted that in two separate experiments (Table IV), the efficacy of free doxorubicin in increasing survival time was markedly different, in contrast to the reproducible effect of the polymeric derivative. Thus, no definitive conclusions could be drawn from these experiments. Relevant to this point is the observation that in a variety of experimental models, reproducible results were generally obtained using different preparations of the conjugate. This also reflects the stability of the conjugate in aqueous solutions.

Taken together with previous results showing an enhanced efficacy of the polymeric derivative as compared to free doxorubicin against leukaemia models (Zunino *et al.*, 1984), the data presented here suggest a therapeutic advantage of the drug linked to PAA in tumours of tissues of mesenchymal origin. Anyway, the attachment of doxorubicin to this macromolecular carrier did not cause loss or reduction of anti-tumour efficacy at optimal doses in any of the tumour models used. However, this means of drug delivery is not expected to overcome drug resistance, since free and polymer-linked doxorubicin produced a marginal response against colon carcinoma 26 (not shown), a tumour model, which, when growing subcutaneously, is relatively resistant to anthracycline therapy (Casazza *et al.*, 1983). This is in agreement with the absence of appreciable activity of the conjugate against a doxorubicin-resistant subline of P388 leukaemia (Zunino *et al.*, 1982).

Finally, it remains to be clarified whether the PAA conjugate has further therapeutic advantage over free drug, when administered in an optimal schedule. It should be noted that in all experiments only optimal schedules of free drug were employed.

In conclusion, among the favourable properties found for the doxorubicin-PAA conjugate in this and previous studies, the following are of particular interest: (i) reduced severity of some specific toxic effects; (ii) increased effectiveness against all of the leukaemia and sarcoma models tested; (iii) anti-tumour activity fully retained in other experimental tumour systems and (iv) improvement of the therapeutic index, more evident in the treatment of highly sensitive tumours (Gross leukaemia [Zunino *et al.*, 1984] and M5 reticulum cell sarcoma [Table VI]).

Further studies on the preclinical toxicity of this polymeric derivative are needed to fully evaluate the therapeutic potential of this drug delivery system.

This work was supported in part by a research grant from the Consiglio Nazionale delle Ricerche, Rome (Progetto Finalizzato Oncologia). We thank Ms B. Johnston for editorial assistance and manuscript preparation.

### References

ARNON, R. & HURWITZ, E. (1983). Antibody- and polymer-drug conjugates. In *Targeted Drugs*, Goldberg (ed) p. 23. John Wiley & Sons: New York.

BERTAZZOLI, C., BELLINI, O., MAGRINI, U. & TOSANA, M.G. (1979). Quantitative experimental evaluation of adriamycin cardiotoxicity in the mouse. *Cancer Treat. Rep.*, **63**, 1877.

CASAZZA, A.M., SAVI, G., PRATESI, G. & DI MARCO, A. (1983). Antitumor activity in mice of 4′-deoxydoxorubicin in comparison with doxorubicin. *Eur. J. Cancer Clin. Oncol.*, **19**, 411.

DI MARCO, A., LENAZ, L., CASAZZA, A.M. & SCARPINATO, B.M. (1972). Activity of adriamycin (NSC-123127) and daunomycin (NSC-82151) against mouse mammary carcinoma. *Cancer Chemother. Rep.*, **56**, 153.

GERAN, R.I., GREENBERG, N.H., MACDONALD, M.M., SCHUMACHER, A.M. & ABBOTT, B.J. (1972). Protocols for screening chemical agents and natural products against animal tumors and other biological systems (third edition). *Cancer Chemother. Rep.*, **3**, 1.

GHOSE, T., BLAIR, A.H., VAUGHAN, K. & KULKARNI, P. (1983). Anti-body-directed drug targeting in cancer therapy. In *Targeted Drugs*, Goldberg (ed) p. 1. John Wiley & Sons: New York.

GREGORIADIS, G. (1981). Targeting of drugs: implications in medicine. *Lancet*, **ii**, 241.

GROS, L., RINGSDORF, H. & SCHUPP, H. (1981). Polymeric antitumor agents on a molecular and on a cellular level? *Angew. Chem.*, **20**, 305.

JENKINS, J. & CORDEN, B.J. (1983). Vesicant activity of chemotherapeutic agents. *Cancer Treat. Rep.*, **67**, 409.

KAYE, S.B. (1981). Liposomes: problems and promise as selective drug carriers. *Cancer Treat. Rev.*, **8**, 27.

LENAZ, L. & PAGE, J.A. (1976). Cardiotoxicity of adriamycin and related anthracyclines. *Cancer Treat. Rev.*, **3**, 111.

LEVI-SCHAFFER, F., BERNSTEIN, A., MESHORER, A. & ARNON, R. (1982). Reduced toxicity of daunorubicin by conjugation to dextran. *Cancer Treat. Rep.*, **66**, 107.

MYERS, C.E. & COLLINS, J.M. (1983). Pharmacology of intraperitoneal chemotherapy. *Cancer Invest.*, **1**, 395.

TALMADGE, J.E., DONOVAN, P.A. & HART, I.R. (1982). Inhibition of cellular division of a murine macrophage tumor by macrophage-activating agents. *Cancer Res.*, **42**, 1850.

TALMADGE, J.E., KEY, M. & HART, I.R. (1981). Characterization of a murine ovarian reticulum cell sarcoma of histiocytic origin. *Cancer Res.*, **41**, 1271.

TARNOWSKI, G.S., RALPH, P. & STOCK, C.C. (1979). Sensitivity to chemotherapeutic and immunomodulating agents of two mouse lymphomas and of a macrophage tumor. *Cancer Res.*, **39**, 3964.

ZUNINO, F., GIULIANI, F., SAVI, G., DASDIA, T. & GAMBETTA, R. (1982). Antitumor activity of daunorubicin linked to poly-L-aspartic acid. *Int. J. Cancer*, **30**, 465.

ZUNINO, F., SAVI, G., GIULIANI, F., GAMBETTA, R., SUPINO, R., TINELLI, S. & PEZZONI, G. (1984). Comparison of antitumor effects of daunorubicin covalently linked to poly-L-amino acid carriers. *Eur. J. Cancer Clin. Oncol.*, **20**, 421.